# Cells respond to deletion of *CAV1* by increasing synthesis of extracellular matrix

**DOI:** 10.1371/journal.pone.0205306

**Published:** 2018-10-22

**Authors:** C. Mendoza-Topaz, G. Nelson, G. Howard, S. Hafner, P. Rademacher, M. Frick, B. J. Nichols

**Affiliations:** 1 Medical Research Council Laboratory of Molecular Biology, Cambridge, United Kingdom; 2 Institute of Pathophysiological Anesthesiology and Process Engineering, University of Ulm, Ulm, Germany; 3 Institute of General Physiology, University of Ulm, Ulm, Germany; University of British Columbia, CANADA

## Abstract

A range of cellular functions have been attributed to caveolae, flask-like invaginations of the plasma membrane. Here, we have used RNA-seq to achieve quantitative transcriptional profiling of primary embryonic fibroblasts from caveolin 1 knockout mice (*CAV1-/-* MEFs), and thereby to gain hypothesis-free insight into how these cells respond to the absence of caveolae. Components of the extracellular matrix were decisively over-represented within the set of genes displaying altered expression in *CAV1-/-* MEFs when compared to congenic wild-type controls. This was confirmed biochemically and by imaging for selected examples. Up-regulation of components of the extracellular matrix was also observed in a second cell line, NIH-3T3 cells genome edited to delete *CAV1*. Up-regulation of components of the extracellular matrix was detected *in vivo* by assessing collagen deposition and compliance of *CAV1-/-* lungs. We discuss the implications of these findings in terms of the cellular function of caveolae.

## Introduction

Caveolae are flask-shaped invaginations of the plasma membrane. They are particularly abundant in endothelial cells, adipocytes, muscle cells, and in specific epithelia including type I alveolar cells of the lung [1, 2]. The molecular components responsible for generating caveolae are increasingly well-characterised, and include: 1. Caveolin proteins, which behave biochemically as integral membrane proteins and form defined oligomers in the inner leaflet of the plasma membrane [3–5], 2. Cavin proteins, which are more soluble than caveolins and form oligomers characterised by the assembly of trimeric coiled coils [6–8], 3. EHD (Eps15 Homology Domain) proteins, which act at the constricted neck of caveolae [9–12], 4. Pacsin 2 (Syndapin 2), which is also present at the neck of at least a subset of caveolae [13, 14]. Caveolins and cavins assemble into a large 80S complex with the size and shape of individual caveolae [15, 16], and both caveolin 1 (the product of the *CAV1* gene) and cavin 1 are essential for formation of caveolae [17–19].

*CAVIN1* and *CAV1* knockout mice, and human patients with rare loss-of-function mutations to caveolar components, display a complex range of phenotypes that suggest important roles for caveolae in maintenance of correct cell function in vascular endothelia, muscle, and adipose tissue [2–5]. The mechanisms underlying these phenotypes are incompletely understood. Caveolae have been proposed to regulate a wide variety of signalling events, including signalling to modulate eNOS activity and signalling to modulate insulin receptor activity [20–22]. Caveolae may bud from the plasma membrane to mediate trans-endothelial vesicular trafficking, or other types of endocytosis [23–25]. Phenotypes of invertebrates where caveolin genes have been deleted suggest functions linked to lipid trafficking or homeostasis, and it is possible that such functions are conserved in mammals [26–28].

Increasing evidence links caveolae to protection of cells from mechanical damage [29–31], via three non-exclusive potential mechanisms: 1. Caveolae introduce folds or convolutions into the plasma membrane, and flattening out of such convolutions when mechanical tension is imposed upon the membrane may buffer tension forces and hence decrease the likelihood of rupture [32, 33], 2. Mechanical cues may elicit intra-cellular signals via caveolae and thereby trigger transcriptional or other adaptive responses [2, 32], 3. Caveolae may be important for the internalisation of damaged membrane regions and hence form part of a membrane repair mechanism [34, 35].

Given the perplexing array of possible cellular functions attributed to caveolae, there is evident utility in detailing precisely how cells respond to the absence of these structures. Such data will provide a hypothesis-free profile of those aspects of the function of individual cells that are most affected by caveolae. Here, we have used RNA-seq to achieve quantitative transcriptional profiling of primary embryonic fibroblasts from caveolin 1 knockout mice (*CAV1-/-* MEFs). We conclude that cells detect the absence of caveolae and respond by producing more extracellular matrix, and discuss the implications of these findings in terms of the cellular function of caveolae.

## Results

Heterozygous *CAV1+/-* mice were crossed to produce wild-type (WT, *CAV1+/+*) and *CAV1-/-* progeny. WT and *CAV1-/-* progeny of the same genotype were bred and MEFs isolated at 13.5 days gestation. In each biological replicate embryos were isolated from four pregnant females and the resultant MEFs pooled. Four separate biological replicates were performed. All replicates were subjected to whole genome RNA sequencing (RNA-seq) analysis after 24–48 hours in primary culture. Among the 39707 genes to which the sequenced reads were aligned, 16420 (41%) were defined as “expressed” with their expression levels higher than 1.0 Fragments Per Kilobase of exon per Million fragments mapped (FPKM). Among expressed genes, we found 103 protein-encoding genes that were differentially expressed between the two genotypes at high statistical confidence (q < 0.05 [36, 37]).

**Fig 1:**
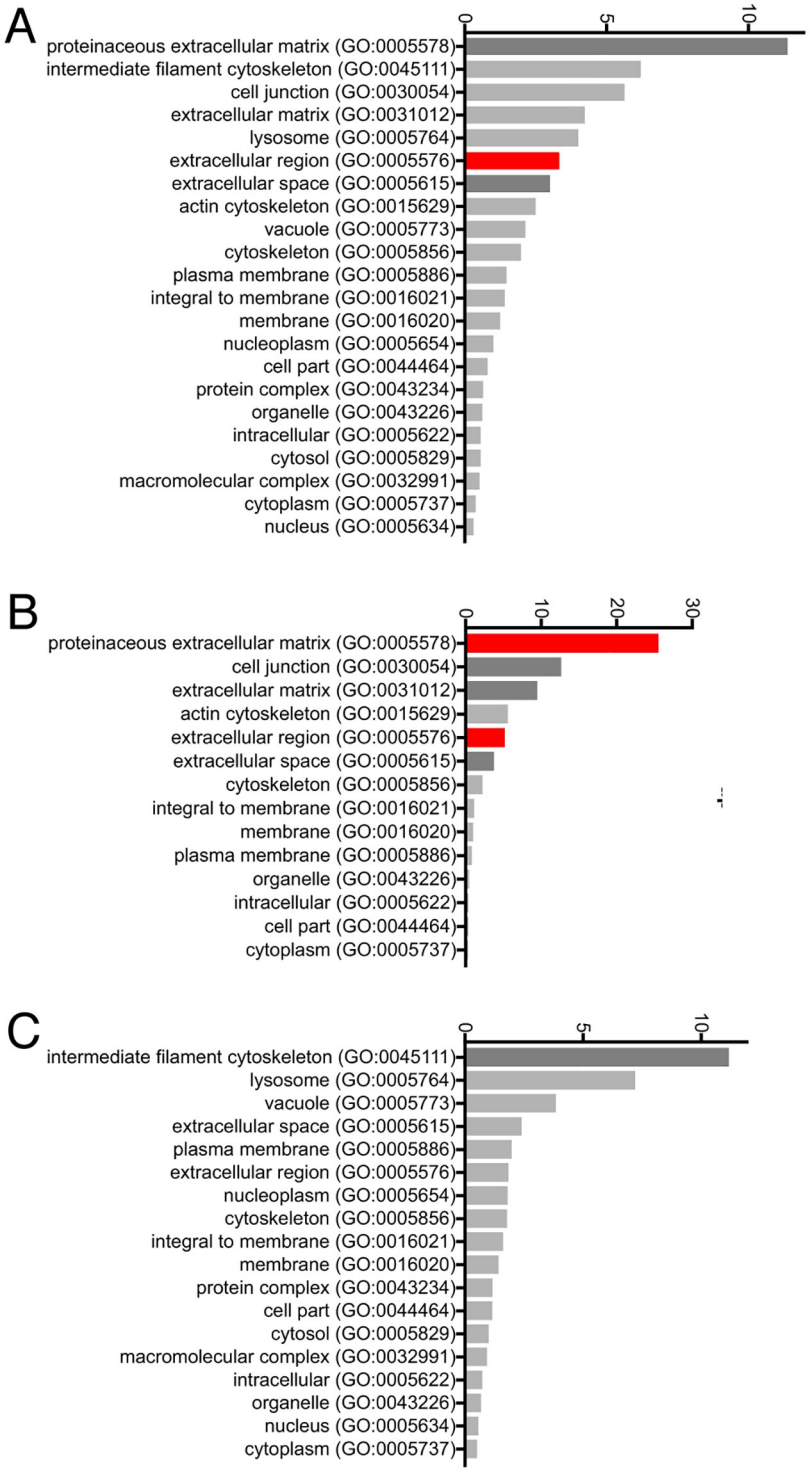
Gene ontology analysis of genes up-regulated and down-regulated in *CAV1-/-* MEFS. **A**. All genes showing significantly (q < 0.05) altered transcript levels were submitted to the Panther gene ontology database to classify their sub-cellular distributions. The number of genes identified in each sub-cellular location is shown. Sub-cellular locations over-represented at P < 0.1 using Bonferroni’s correction for multiple testing are shaded darker grey, and at P < 0.01 are shaded red. **B**. As A but only up-regulated genes were analysed. **C**. As A but only down-regulated genes were analysed.

Lists of translated genes identified as expressed differently between WT and *CAV1-/-* MEFs with high statistical confidence, along with the amplitude of the change for each gene, are shown in Tables 1 and 2 (Full dataset is in S1 File). The Panther gene classification system (www.pantherdb.org) was used to classify the sub-cellular location of the gene products using previously determined ontology (Fig 1A).

**Table 1:** List of genes significantly up-regulated in *CAV1-/*- MEFS. Only protein-encoding genes where q < 0.05 are shown, the full RNA-seq dataset is contained in S1 File. N = 4 biological replicates, each individual replicate comprising analysis of RNA pooled from all embryos from four mice.

	UP in *CAV1-/-*	
	log2(fold change)	q value
**Rpsa-ps10**	-8.37741	0.00692166
**Boll**	-3.9005	0.00692166
**Hp**	-2.6409	0.00692166
**Cxcl13**	-2.56011	0.00692166
**Map4k2**	-2.00809	0.0375919
**Col6a6**	-1.63478	0.00692166
**Tnn**	-1.51824	0.00692166
**Prelp**	-1.47382	0.00692166
**Dpt**	-1.39127	0.00692166
**Acp5**	-1.20669	0.0176127
**Sfrp2**	-1.20043	0.0340759
**Il33**	-1.19136	0.0262263
**Ntrk2**	-1.06095	0.00692166
**Col28a1**	-1.04536	0.0220741
**Galnt16**	-1.00456	0.00692166
**Gdf10**	-0.998608	0.00692166
**Dcx**	-0.957355	0.00692166
**Tril**	-0.955059	0.00692166
**Mapt**	-0.936393	0.0302349
**Egfl6**	-0.896372	0.0375919
**Wisp2**	-0.885063	0.0442584
**Fat4**	-0.870303	0.00692166
**Fzd4**	-0.869915	0.00692166
**Abcc9**	-0.869901	0.0262263
**Tbxa2r**	-0.853815	0.0262263
**Brd3**	-0.842777	0.00692166
**Itih5**	-0.83026	0.0126897
**Adamts15**	-0.829148	0.0375919
**Igfbp3**	-0.805513	0.0126897
**Fbn2**	-0.795042	0.00692166
**Parm1**	-0.793557	0.00692166
**Fras1**	-0.786727	0.00692166
**Dner**	-0.78094	0.00692166
**Tmem26**	-0.765602	0.0302349
**Ror1**	-0.746148	0.0176127
**Fmod**	-0.737573	0.0176127
**Dcn**	-0.730383	0.0262263
**Sema6a**	-0.723499	0.0408339
**Myh11**	-0.715594	0.00692166
**Prss35**	-0.685936	0.0442584
**Cdkn1c**	-0.681699	0.00692166
**Myh3**	-0.680278	0.0176127
**Actn2**	-0.671471	0.0375919
**Sfrp1**	-0.640639	0.0176127
**Lrrc15**	-0.610529	0.0126897
**Mylk**	-0.560148	0.0302349

**Table 2:** List of genes significantly down-regulated in *CAV1-/*- MEFS. Only protein-encoding genes where q < 0.05 are shown, the full RNA-seq dataset is contained in S1 File. N = 4 biological replicates, each individual replicate comprising analysis of RNA pooled from all embryos from four mice.

	DOWN in CAV1-/-	
	log2(fold change)	q value
**Cav1**	8.81982	0.00692166
**Pde12**	6.49034	0.00692166
**Cacng8**	3.93173	0.00692166
**Trac**	3.51202	0.00692166
**Gria2**	2.85064	0.00692166
**Cck**	2.79191	0.00692166
**Rcan3**	2.72496	0.00692166
**Rpph1**	2.67796	0.00692166
**Dnmt3l**	2.5517	0.0474348
**Gfra4**	2.24171	0.00692166
**Nqo1**	2.24081	0.00692166
**Serpinb9b**	1.74591	0.00692166
**Zfp518a**	1.73215	0.00692166
**Snhg11**	1.69057	0.0262263
**Ccl12**	1.68409	0.0176127
**Lars2**	1.66528	0.00692166
**Gsta4**	1.64305	0.00692166
**Cbr3**	1.53192	0.0126897
**Josd2**	1.52855	0.00692166
**Sncg**	1.37951	0.0220741
**Spta1**	1.34629	0.0176127
**Esm1**	1.29934	0.00692166
**Pparg**	1.2983	0.0176127
**Ptgs1**	1.28854	0.00692166
**Procr**	1.2688	0.0375919
**Ly6a**	1.22294	0.00692166
**Pf4**	1.20743	0.0176127
**Cldn6**	1.1319	0.0408339
**Flt4**	1.11217	0.0474348
**Glce**	1.09499	0.00692166
**Cobll1**	1.08438	0.00692166
**Gng11**	1.08214	0.00692166
**Krt17**	1.05113	0.0262263
**Cldn4**	0.962702	0.0176127
**Slc14a1**	0.929073	0.0302349
**Hmga1**	0.883036	0.00692166
**F13a1**	0.88052	0.0340759
**Neat1**	0.870758	0.00692166
**Clca1**	0.865692	0.0220741
**Grem1**	0.831465	0.00692166
**Esd**	0.828259	0.00692166
**Prkar2b**	0.824013	0.0262263
**Cd34**	0.820626	0.00692166
**Plaur**	0.792514	0.00692166
**Tspo**	0.777008	0.0126897
**Csf1**	0.768798	0.0340759
**S100a6**	0.765292	0.00692166
**Gprc5a**	0.745829	0.0262263
**Ccnd1**	0.734986	0.00692166
**Krt8**	0.719882	0.0220741
**Hspb8**	0.677081	0.0126897
**Dusp4**	0.674591	0.0220741
**Cd151**	0.656597	0.0220741
**Tinagl1**	0.65022	0.0302349
**Snai1**	0.634039	0.0176127
**Cdkn1a**	0.626359	0.0176127
**Tm4sf1**	0.601498	0.0375919

The only location terms that were significantly over-represented in this set were *proteinaceous extracellular matrix*, *extracellular region* and *extracellular space*. When only those genes up-regulated in *CAV1-/-* MEFs were analysed, *proteinaceous extracellular matrix*, *cell junction*, *extracellular matrix*, *extracellular region* and *extracellular space* were all significantly over-represented, and indeed these terms applied to 22 out of 46 up-regulated genes (Fig 1B). When only down-regulated genes were analysed there was less of an obvious overall pattern, though genes with the term *intermediate filament cytoskeleton* were over-represented (Fig 1C). One clear conclusion from our RNA-seq analysis is, therefore, that transcripts for extracellular matrix (ECM) components are up-regulated in *CAV1-/-* MEFs.

**Fig 2:**
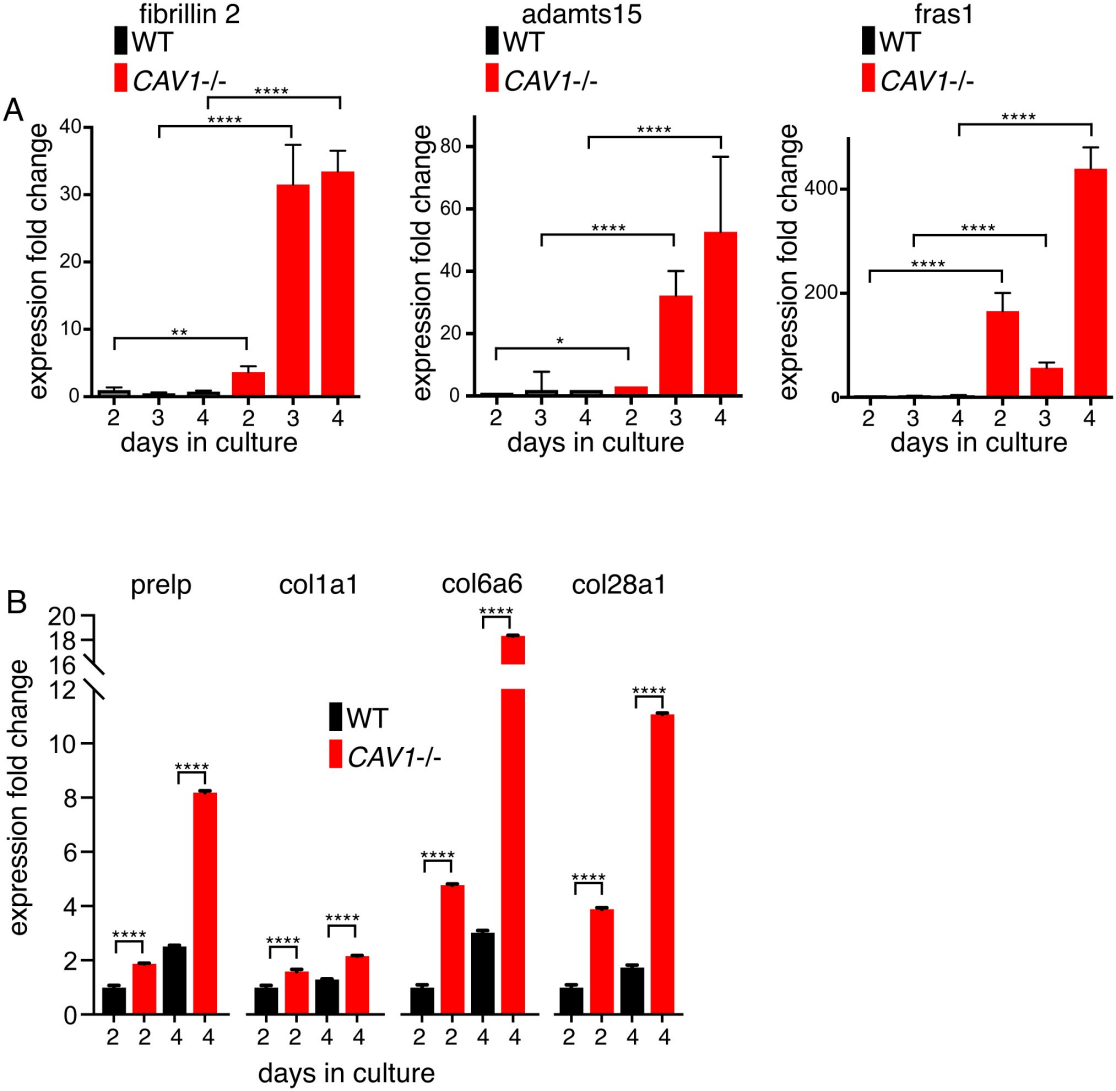
qPCR of selected *CAV1*-regulated mRNAs confirms up-regulation of ECM transcription. **A**. Quantitative PCR was used to measure the levels of the transcripts shown in mRNAs purified from three different isolates of *CAV1-/-* MEFs (KO) and congenic WT controls. Expression fold change normalised to housekeeping controls is derived from ΔΔCT. The MEFs were grown in culture for the number of days shown. Bars are SD, N = 4 experimental repeats. P values were determined using a T-test. **B**. Quantitative PCR was used to measure the levels of the transcripts shown in mRNAs purified from *CAV1-/-* MEFs (KO) and congenic WT controls. The MEFs were from a single isolate per genotype, and were grown in culture for the number of days shown. Bars are SD, N = 4 experimental repeats. P values were determined using a T-test.

We focussed on expression of specific ECM components identified as up-regulated, and used quantitative PCR to confirm the changes in expression detected by RNA-seq. PCR analysis of RNA isolated from new MEF preparations confirmed that message levels for fibrillin 2 (which forms elastic microfibrils within the ECM [38]), adamts15 (a matrix-associated peptidase [39]), and FRAS1 (Fraser extracellular matrix complex 1 –an ECM component abundant in basement membrane [40]), are all highly up-regulated in *CAV1-/-* MEFS (Fig 2A).

Noting that the expression of these ECM components tends to increase with time after plating of the cells, we compared expression of further ECM components at two and four days after plating in WT and *CAV1-/-* MEFS. Expression of PRELP (proline/arginine-rich end leucine-rich repeat protein, a component of connective tissues [41]), and three collagen variants (collagen 6a6and collagen 28a1 which were identified as having increased expression in our RNA-seq data set, and collagen1a1 which is a widely expressed component of abundant fibrillar collagen) all followed the same pattern of increased mRNA levels with time after plating, and all showed considerably higher mRNA levels in the *CAV1-/-* cells than controls (Fig 2B). mRNA levels for multiple ECM components are clearly increased in *CAV1-/-* MEFs.

**Fig 3:**
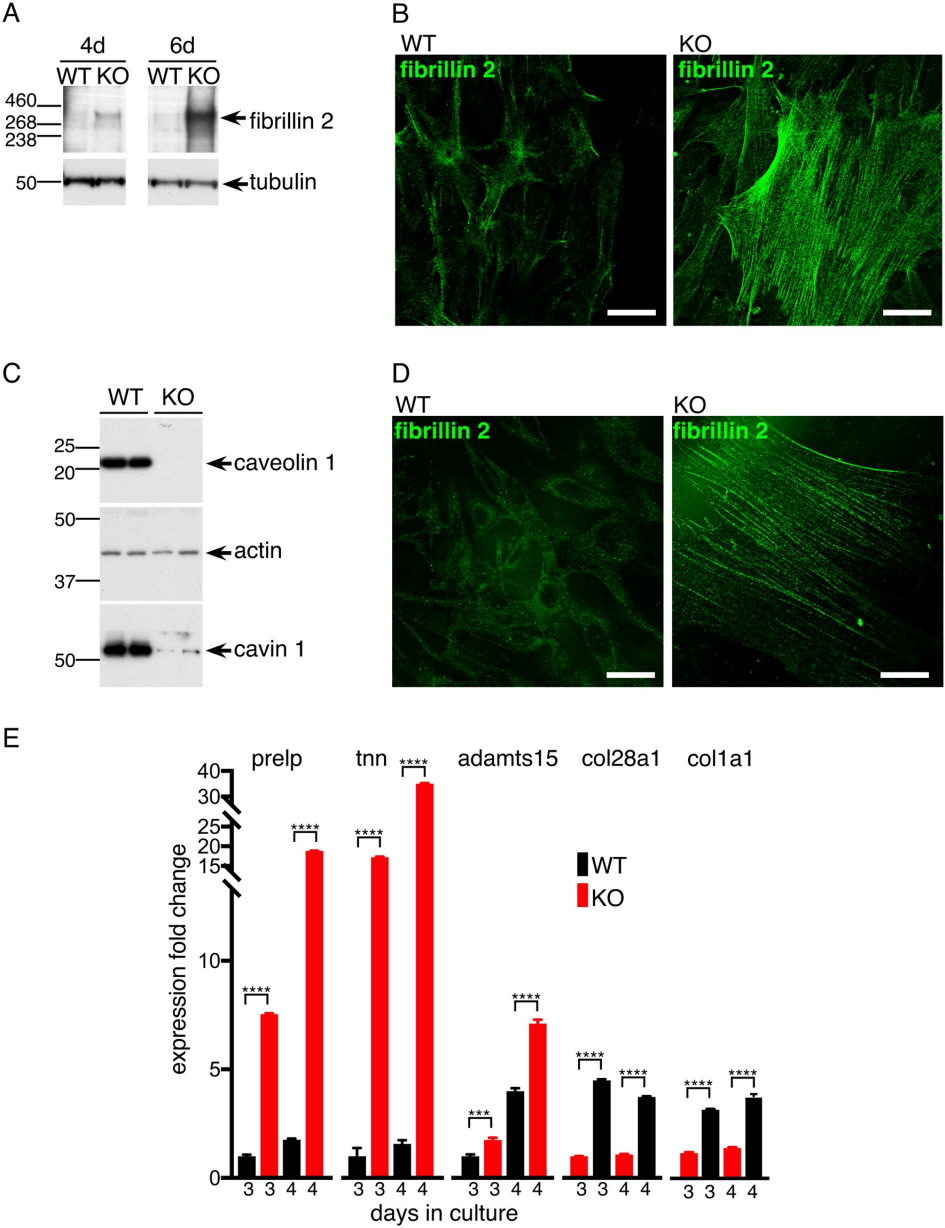
Fibrillin2 protein levels are increased in *CAV1-/-* MEFS and *CAV1* KO NIH-3T3 cells. **A.** Western blots with anti-fibrillin-2 or anti-tubulin antibodies of lysates from *CAV1-/-* MEFs (KO) and congenic WT controls harvested at the indicated number of days after plating. **B**. Indirect immunofluorescence with anti-fibrillin-2 antibodies labelling either *CAV1-/-* MEFs (KO) or congenic WT controls fixed and stained four days after plating. Bars 10μ. **C**. Western blots to demonstrate absence of caveolin 1 in CRISPR-generated *CAV1* knockout NIH3T3 cells. Absence of caveolin1 results in reduced expression of cavin1. **D**. Indirect immunofluorescence with anti-fibrillin-2 antibodies labelling either control NIH3T3 cells (WT) or *CAV1* null NIH3T3 cells (KO). Bars 10μ. **E**. Quantitative PCR was used to measure the levels of the transcripts shown in mRNAs purified from *CAV1 KO* NIH-3T3 cells and WT controls. Expression fold change is relative to the WT, 3 days culture sample. The cells were grown in culture for the number of days shown. Bars are SD, N = 4 experimental repeats. P values were determined using a T-test.

Fibrillin 2 was selected for further analysis, as the assembly of this protein into characteristic microfibrils facilitates unambiguous identification of specific signal after labelling by indirect immunofluorescence [42, 43]. In order to ascertain whether increased mRNA levels do indeed result in increased protein expression, lysates from WT and *CAV1-/-* MEFS were analysed by Western blotting with anti-fibrillin antibodies (Fig 3A).

When cells were harvested four days after plating, a clear difference between WT and *CAV1-/-* was detected, and this difference increased with time (Fig 3A). In complementary experiments, WT and *CAV1-/-* MEFs were stained by indirect immunofluorescence with anti-fibrillin antibodies at four days after plating. Characteristic and prominent microfibrils were prominent in the *CAV1-/-* MEFs, but were much less abundant in the WT cells (Fig 3B).

It was possible that the increase in ECM component synthesis described above is specific to primary MEFs. Application of CRISPR-based genome editing to produce *CAV1* knockout (KO) NIH-3T3 cells allowed us to ask whether similar results were obtained in a second cell type (Fig 3C, and S1 Fig). WT and *CAV1* KO NIH-3T3 cells were stained by indirect immunofluorescence with anti-fibrillin antibodies. Again, microfibrils were much more prominent in the *CAV1* KO NIH-3T3 cells (Fig 3D).

The *CAV1* KO NIH-3T3 cells described above also allowed us to test whether a wider range of ECM components are up-regulated in a second cell type. Quantitative PCR showed that message levels of multiple transcripts revealed by RNASeq and quantitative PCR to be up-regulated in *CAV1*-/- MEFs are also up-regulated in the *CAV1* KO NIH-3T3 cells (Fig 3E). Additional transcripts, such as collagen 6a6, were not detected in the WT NIH-3T3 cells but were detected in the *CAV1* KOs, so despite the fact that a fold-change of expression of this transcript could not be calculated, it is clearly highly up-regulated when caveolin 1 is absent (not shown).

**Fig 4:**
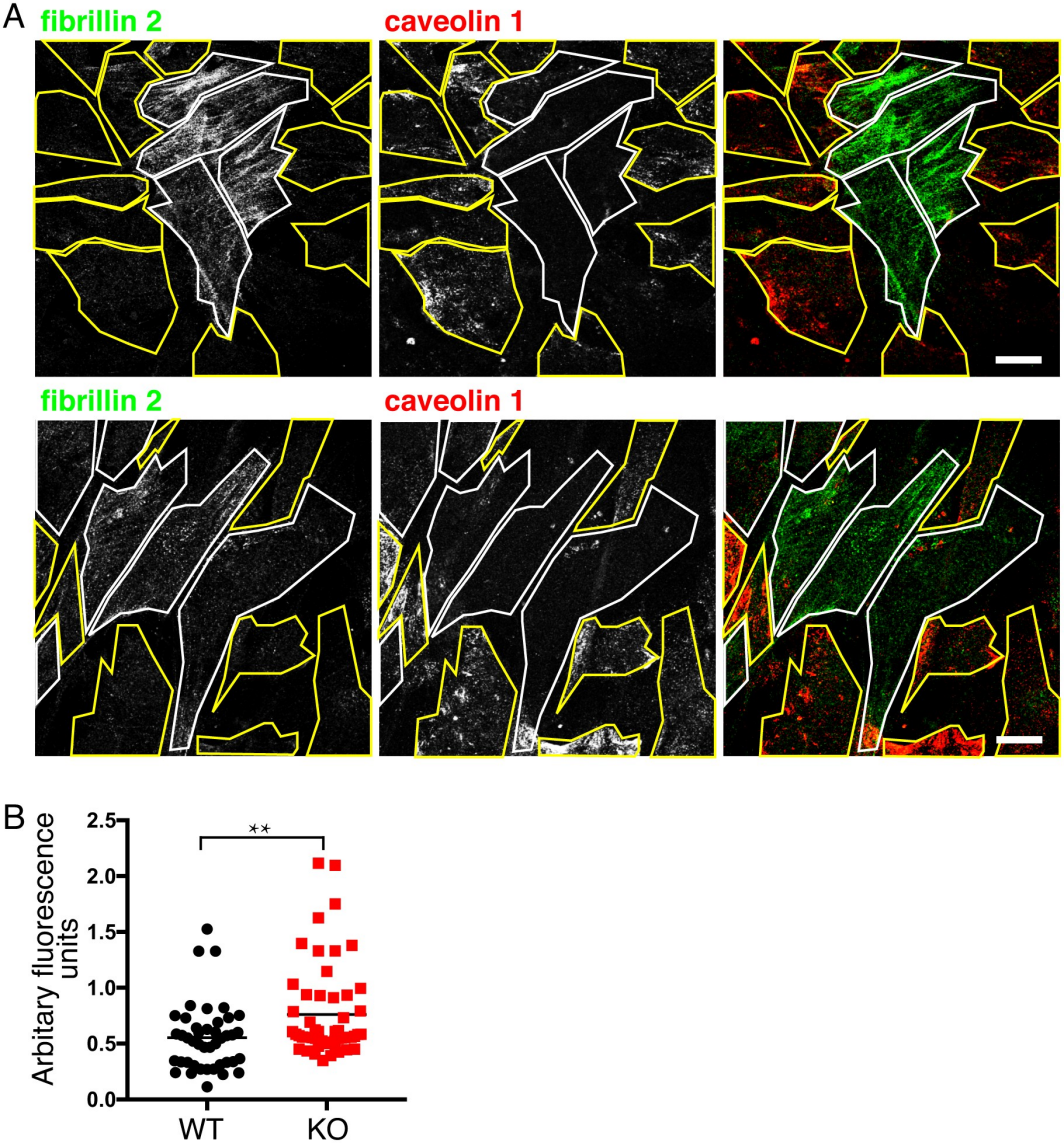
Increased fibrillin 2 expression in *CAV1-/-* MEFS is maintained during co-culture with WT MEFs. **A**. Indirect immunofluorescence with anti-fibrillin-2 and anti-caveolin-1 antibodies labelling *CAV1-/-* MEFs (KO) and congenic WT controls plated as a mixed culture for 10 days. Two representative fields of cells are shown, KO cells identified by absence of caveolin 1 signal are outlined in white, WT cells are outlined in yellow. Bars 10μ**. B**. Quantification of fibrillin 2 expression in WT and *CAV1-/-* MEFs plated as mixed cultures as in A, expressed in arbitrary fluorescence units from the mean fluorescence intensity of individual cell areas after background subtraction. P value was determined using a T-test.

We considered two possible types of mechanism that could both cause increased production of ECM components in *CAV1-/-* cells. Pro-inflammatory cytokines or other signals could be released from the cells in response to stress / damage, and there could also be a mechanism integral to individual cells for sensing and reacting to changes caused by the absence of caveolae. We addressed these possibilities by culturing WT and *CAV1-/-* MEFs as mixed cultures for ten days. Cells were stained with anti-caveolin 1 and anti-fibrillin antibodies (Fig 4A and 4B).

Microfibrils were clearly much more prominent on the *CAV1-/-* cells (Fig 4A and 4B). This was consistently the case in cells plated at different ratios between genotypes and co-cultured for different times. We did not observe large differences in doubling times in cells of different genotypes. These data provide a preliminary indication that, in this case, release of pro-inflammatory or other signals may be less important than an intrinsic mechanism in triggering the observed increase in ECM component production.

**Fig 5:**
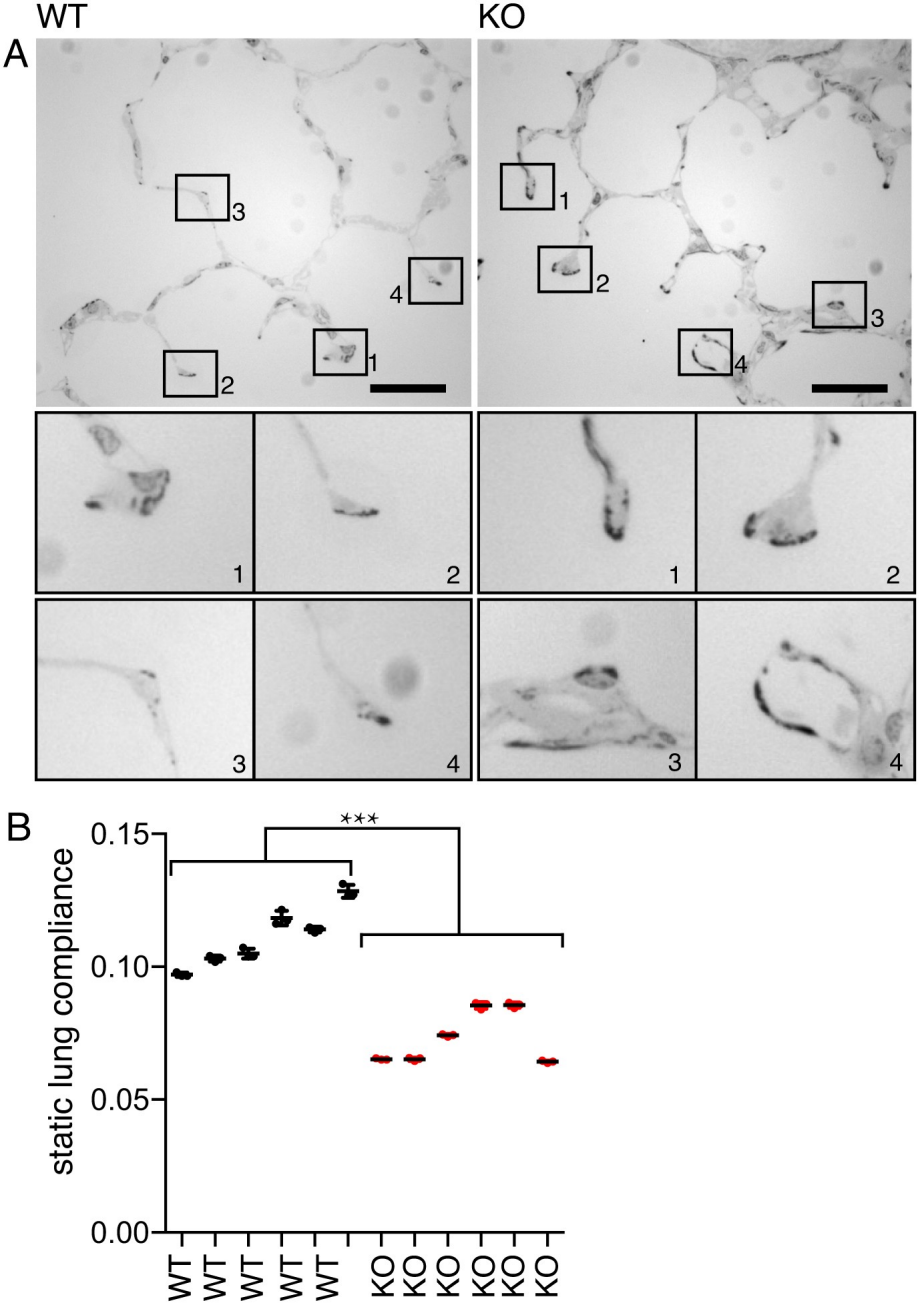
Fibrosis and reduced compliance in lungs from *CAV1-/-* mice. **A.** Epoxy-embedded lung sections stained with phloxine B and azure blue. Bars 20μ. The lower panels show magnified images of the tips of septae where increased staining is evident in the *CAV1-/-* samples. **B**. Lung compliance in WT and CAV1-/- lungs. Each column represents measurements from an individual mouse, the measurement was repeated four times per individual. Lines are mean and SD. P value was determined using a T-test.

Increased ECM production by *CAV1-/-* cells in culture suggests that similar changes should occur in tissues of *CAV1-/-* mice, and that these changes may underlie some of the complex phenotypes displayed by these mice [18]. We stained epoxy-embedded lung sections with phloxine B and azure blue, which emphasises the elastin and collagen fibre bundle elements of the ECM [44]. As predicted by the results presented above, staining was much more prominent in samples from *CAV1-/-* mice than from the WT controls (Fig 5A).

So as to ascertain whether increased ECM production has an effect on the properties of the lung we measured compliance of lungs dissected from congenic, age-matched WT and *CAV1-/-* mice (Fig 5B). Knockout lungs were significantly less compliant when inflated at equivalent pressures. Although a complex set factors may influence lung compliance, there is good evidence that elastin and collagen levels are critical parameters [45, 46]. Our data are therefore consistent with the increased ECM production in *CAV1-/-* mice having a direct impact on lung function.

## Discussion

Our data lead to two main conclusions. First, and most importantly, we show that cells respond to lack of caveolin 1 by producing more extracellular matrix components.

Second, experiments measuring lung compliance and ECM deposition show that increased ECM production may underlie some of the diverse and hard-to-explain set of phenotypes reported for mice and rare human patients that lack caveolae due to mutations in *CAV1* or *CAVIN1*. Fibrosis of multiple tissues has already been reported in *CAV1* null animals [18, 27, 28, 47, 48]. *CAVIN1* knockout mice also show fibrosis and altered elastic properties of the lung [49]. Levels of inflammatory cytokines are elevated in the lungs these mice, so inflammatory responses are clearly likely to be important *in vivo* [49]. Our data, however, suggest that fibrotic phenotypes are not only caused by release of cytokines or other cell-damage signals. Mechanisms intrinsic to individual cells may also be important.

The levels of cavin 1 and caveolin 1 proteins are co-dependent, as reduction in the levels of caveolin 1 causes a reduction in cavin 1 and *vice versa*. Reduction in caveolin 1 protein also affects the expression levels of other cavin proteins [6, 50–53]. Therefore the sequence of molecular events by which deletion of *CAV1* causes alterations in ECM expression could include changes in the level of caveolar proteins additional to caveolin 1 itself. Also, it is important to note that it is possible that caveolin 1 may have functions outside of morphologically-defined caveolae, and such still-to-be-defined functions could impact on ECM expression [54, 55].

The finding that the most prominent general response of cells to the absence of functional *CAV1*, and hence caveolae, is to produce more ECM, informs the on-going debate as to the central function of caveolae. If the central function of caveolae is to protect cells from mechanical stress at the plasma membrane, then production of more ECM can readily be rationalised as a compensatory process to minimise such stress. This does not, however, rule out alternative explanations and functional models. Notably, analysis of altered gene expression in tissues and starved cells from *CAV1*-/- mice using a gene-chip hybridisation approach revealed that several genes involved in lipid metabolism are expressed at altered levels in these mice relative to controls [56]. Our RNA-seq data do not reveal such changes, and it may be that the changes in lipid metabolism seen *in vivo* are contingent on complex interplay between adipocyte and liver metabolism that are not recapitulated in MEFs in culture [57]. In addition, there are data linking caveolae to endocytosis, and changes in rates of endocytosis of ECM components could be involved in the effects that we report here [31, 58].

It will be important for future experiments to determine the mechanisms underlying increased ECM deposition in the absence of caveolae. Cavin1 has been proposed to inhibit collagen gene expression by associating with a protein called binding factor of a type-1 collagen promoter (BFCOL1) [28, 59], and this could explain increased expression of collagens in *CAV1*-/- cells, as cavin1 protein levels are reduced in these cells [50]. However, it is not clear that BFCOL1 regulates expression of all of the wide range of ECM components that show altered mRNA levels in our dataset. If increased ECM production is indeed a way to minimise tension forces within the membrane, then our data imply the existence of a signalling pathway to detect increased forces in the absence of caveolae and to regulate ECM synthesis accordingly. The molecular details of such a signalling pathway are not at all clear.

## Methods

### Animal procedures

All experiments using mice were conducted under a UK Home Office license, and were approved by the Ethical Review Committee of the Medical Research Council, Laboratory of Molecular Biology. *CAV1*-/- mice have the first two exons of the CAV1 gene deleted [17]. We have backcrossed these mice onto the C57BL/6J background for more than seven generations [50]. To obtain the matching littermates, 15 breeding pairs of heterozygote *CAV1-/+* mice were set up, and control (+/+) and *CAV1* null (-/-) mice were selected from the progeny and used for the timed mating experiments. The pups for the preparation of primary mouse embryonic fibroblasts (MEF) were obtained from those timed mating crosses.

### MEF culture

Primary MEFs were obtained from day 13.5 embryos. Briefly, after lethal injection of sodium pentabarbitone, day E13.5 embryos from timed mating crosses were collected, decapitated, and siblings were pooled (between 5–11 pups per pregnant female). Tissue was thoroughly minced and cells were dissociated with approximately 2 ml of 0.25% trypsin in EBSS per embryo. After 10 min incubation at 37°C without shaking or agitation, cell suspension was removed slowly and placed in new tube and centrifuged 5 min at 1000 rpm. Cell pellet was resuspended in full medium (1 ml per pup) [Dulbecco’s modified Eagle’s medium (DMEM) supplemented with 10% fetal bovine serum (FBS), 2 mM glutamine, 50 U/mL penicillin—streptomycin (Gibco)] and 1ml of the cell suspension was added per 10 cm tissue culture dish with 10 ml full medium. Cells were incubated overnight, medium changed after 24 hrs and incubated for another 1 or 2 days until confluence, when RNA was extracted.

### RNA extraction and processing

RNA was extracted using RNeasy mini kit (Qiagen) following manufacturer instructions, DNA was removed with on-column DNaseI digestion (RNase-free DNaseI, Qiagen) and total RNA was analyzed for quality and quantity using an Agilent Bioanalyzer (Agilent RNA Nano chips, Agilent). polyA mRNA was purified and fragmented using poly-T oligo attached magnetic beads in accordance to manufacturer instructions, (Illumina). The cleaved RNA fragments were primed with random hexamers into first strand cDNA followed by reverse transcription using reverse transcriptase (SuperScriptII reverse transcriptase, Invitrogen) and random primers. AgenCourt Ampure XP beads (Beckman) were used to separate the dscDNA from the second strand reaction mix.

### RNA-seq

Sequencing libraries were prepared from amplified cDNA following manufacturer instructions (TruSeq Stranded mRNA LT, Illumina). Briefly, 3’ end were adenylated, adapters were ligated, and PCR enrichment of DNA fragments with adapters on both ends was performed. Libraries were validated by quantification using the KAPA Library Quantification kit (Roche) in an Applied Biosystem Vii7 instrument and quality control was done using an Agilent Bioanalyzer with Agilent DNA chips (Agilent). Indexed libraries were normalized and pooled and sequencing was performed using the HiSeq platform (Illumina) with single-end 50 bp reads at the CRUK Cambridge Institute Genomics Core facility.

### RNA-seq data processing and analysis

Reads were aligned via TopHat2 v2.0.13 [60], to the GRCm38 mouse transcriptome (—no-coverage-search,—library-type = fr-firststrand,—transcriptome-index). The Cufflinks suite v2.2.1 [36, 37] was used to quantify transcripts and also identify differentially expressed genes with *q* < 0.05 (—library-type = fr-firststrand,—frag-bias-correct,—multi-read-correct).

### Quantitative Real-Time PCR and probes

Total RNA was isolated from cells using the RNeasy Mini Kit (Qiaqen) and reverse transcribed using the High-Capacity RNA-to-cDNA Kit (Applied Biosystems). Quantitative PCR analysis was performed using TaqMan probes with FAM-MGB dyes that span exons and and TaqMan Universal Master Mix II, with UNG (Applied Biosystems) on a ViiA7 Real-Time PCR System (Applied Biosystems). Probes used were Mm00515713_m1 (Fnb2), Mm00446968_m1 (Hprt), Mm 99999915_g1 (GAPDH), Mm01129316_m1 (Caveolin1), Mm01176187_m1 (Adamts15), Mm 04212217_m1 (Fras1), Mm01294828_m1 (Prelp), Mm00556810_m1 (Col6a6), Mm00801666_g1 (Col1a1) and Mm01166176_m1 (Col28a1). **ΔΔ**Ct was calculated using relative expression normalized to either GAPDH or Hprt1.

### Statistical analysis

All pairwise comparisons of data are carried out using Student’s T-test. Sample sizes are given in the Figure Legends. P values are shown as follows: P<0.05 *, P<0.01 **, P<0.001 ***, P<0.0001 ****.

### Antibodies

The following antibodies were used: rabbit anti-Caveolin1 (BD Biosciences Cat# 610060), anti Cavin1 (Abcam Cat# ab48824), mouse anti-Fibrillin2 (Santa Cruz Biotechnology H-10 sc-393968), rat YL1-2 anti-alpha tubulin (in-house cell culture supernatant), rabbit anti-actin (Sigma C3956). Horse radish peroxidase (HRP)-conjugated secondary antibodies were from DAKO.

### Immunofluorescence

Cells were fixed at -20°C with 70% MetOH 30% Acetone 5min, blocked 30min with 10% FBS in PBS and stained with anti-Caveolin1 (1:5000) and anti-Fbn2 (1:150) overnight at 4°C. After several washes with PBS, cells were incubated 1hr at room temperature with the appropriate secondary antibodies conjugated with Alexa dyes (Invitrogen) diluted 1:500, washed several times with PBS and mounted in ProLong Gold antifade reagent (Molecular Probes).

### Genome editing

For the generation of NIH3T3 Cav1KO cell line a single sgRNA (5’ CACCGATGTTGCCCTGTTCCCGGAT) was cloned into pSpCas9(BB)-2A-GFP (PX458) plasmid (Addgene plasmid #48138) [61], and then transfected using Neon transfection system (Invitrogen). Caveolin 1 protein was not detected by Western blot with a polyclonal antibody that detects all isoforms of caveolin 1. Sequencing of a PCR product from primers 5’-CGAGGGGTGTGGTGTCCTCCGCTCCG-3’ and 5’-GTGCATGTGTGTGGTGGGGCACTCGTGGC-3’ and genomic DNA from knockout clones show a deletion of 137 bases comprising 37 bases of first intron and 100 bases of exon 2.

### Microscopy

All confocal imaging was carried out using a Zeiss LSM510 inverted confocal microscope with a 63x, 1.4NA objective, driven by Zen software.

### Western blots

Samples were lysed in 1X sample buffer (NuPage Invitrogen) with 150mM DTT, boiled and run on pre-cast 3%–8% Tris-Acetate or 4%-12% Bis-Tris gels (Invitrogen). The gels were then blotted using wet transfer, the membrane blocked in a PBS solution containing 5% dried skimmed milk powder and 0.1% Tween-20, incubated with the appropriate primary antibodies overnight at 4°C, washed with PBS with 0.1% Tween-20 and incubated with HRP conjugated secondary antibodies for 1h room temperature. The blots were developed using Immobilon Western Chemiluminescent HRP Substrate (Millipore) or ECL Western Blot Detection Reagent Kit (GE Healthcare) onto Fuji Super RX X-ray films.

### *In situ* lung compliance measurements

Animals were anesthetized with sevoflurane (2.5%; Sevorane, Abbott). Animals were then euthanized by cervical dislocation and the thorax was surgically opened. Lungs were ventilated via a tracheal catheter using a module 1 flexiVent rodent ventilator (FlexiVent, Scireq). Mechanical ventilation was performed with a pressure-controlled, lung-protective ventilation strategy. Static pulmonary compliance was measured by incrementally increasing the airway pressure up to a maximum inspiratory pressure of 20cm H_2_O and using the indwelling software of a specially designed small animal ventilator (FlexiVent, Scireq) that allows automatic recording of the inspiratory and expiratory pressure–volume loop.

### Phloxine B and azure blue staining

Isolated lungs were fixed and dehydrated with glutaraldehyde, osmium tetroxide, uranyl acetate and ethanol in sequence [50]. Following embedding in Spurr’s epoxy resin 1 micron sections were cut with a microtome. The sections were stained with a 1% aqueous solution of Phloxine B for 1–2 minutes. After washing with distilled water the sections were stained with 1% Azure II and 1% Methylene Blue in 1% borax aqueous solution for 2–5 minutes before mounting on a slide for examination by light microscopy [44].

## Supporting information

S1 Fig Generation of *CAV1*KO NIH3T3 cells. **A.** Gene targetting and PCR genotyping strategy. 2 guide RNAs were employed to delete a region of exon 2 in the *CAV1* gene. Location of PCR primers and products for genotyping are shown in red. **B**. Representative agarose gel showing PCR products from parental WT NIH3T3 cells and from a clone of NIH3T3 cells were caveolin 1 protein is not expressed (Fig 3C).
(PDF)

S1 File All RNAs identified in RNASeq experiments. Data pooled from 4 biological replicates, all 4 biological replicates used to calculate the q values shown.
(XLSX)
