# Supplementary material for: Cells respond to deletion of CAV1 by increasing synthesis of extracellular matrix
Source: PLoS One. 2018 Oct 22;13(10):e0205306. doi: 10.1371/journal.pone.0205306 (PMC6197626; doi:10.1371/journal.pone.0205306)
Supplement: S1 Fig — A. Gene targetting and PCR genotyping strategy. 2 guide RNAs were employed to delete a region of exon 2 in the CAV1 gene. Location of PCR primers and products for genotyping are shown in red. B. Representative agarose gel showing PCR products from parental WT NIH3T3 cells and from a clone of NIH3T3 cells were caveolin 1 protein is not expressed (Fig 3C). (PDF) [file pone.0205306.s001.pdf]

Supporting Figure 1

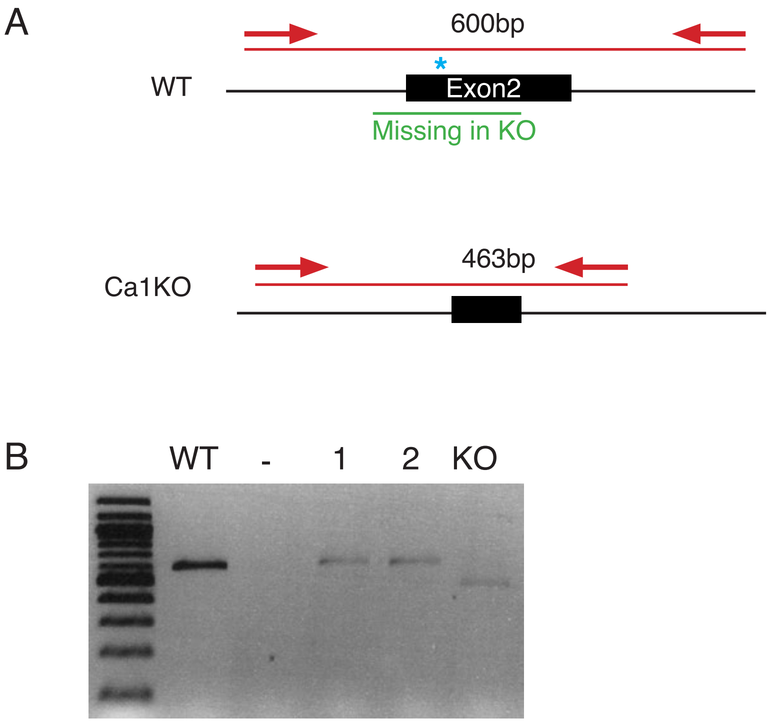

Supporting Figure 1. Generation of *CAV1*KO NIH3T3 cells. A. Gene targetting and PCR genotyping strategy. 1 guide RNAs was employed to delete a region of exon 2 in the *CAV1* gene. Location of PCR primers and products for genotyping are shown in red. **B**. Representative agarose gel showing PCR products from parental WT NIH3T3 cells and from a clone of NIH3T3 cells were caveolin 1 protein is not expressed (Figure 3C).
